# *Crotalus durissus ruruima* Snake Venom and a Phospholipase A_2_ Isolated from This Venom Elicit Macrophages to Form Lipid Droplets and Synthesize Inflammatory Lipid Mediators

**DOI:** 10.1155/2019/2745286

**Published:** 2019-11-04

**Authors:** Ana Eduarda Zulim de Carvalho, Karina Giannotti, Elbio Leiguez Junior, Márcio Matsubara, Maria Cristina Dos Santos, Consuelo Latorre Fortes-Dias, Catarina Teixeira

**Affiliations:** ^1^Butantan Institute, São Paulo, São Paulo, Brazil; ^2^Departamento de Ciências Fisiológicas, Universidade do Amazonas, Manaus, Brazil; ^3^Fundação Ezequiel Dias, Belo Horizonte, Minas Gerais, Brazil

## Abstract

Viper snake *Crotalus durissus ruruima* (Cdr) is a subspecies found in northern area of Brazil. Among the snakes of *Crotalus* genus subspecies, the venom of Cdr presents highest level of crotoxin, which is the major component of *Crotalus* snake venoms, formed by two subunits (crotapotin and a phospholipase A_2_ named CBr) and presents potent neurotoxic activity. Curiously, the venom of *C. d. ruruima* (CdrV) is better neutralized by antibothropic than by anticrotalic serum, strongly suggesting that this venom has similarities with venom of *Bothrops* genus snakes with regard to the ability to induce inflammation. Macrophages are cells with a central role in inflammatory and immunological responses. Upon inflammatory stimuli, these cells exhibit increased numbers of lipid droplets, which are key organelles in the synthesis and release of inflammatory mediators. However, the effects of CdrV and CBr in macrophage functions are unknown. We herein investigated the ability of CdrV and CBr to activate macrophages with focus on the formation of lipid droplets (LDs), synthesis of lipid mediators, and mechanisms involved in these effects. The involvement of LDs in PGE_2_ biosynthesis was also assessed. Stimulation of murine macrophages with CdrV and CBr induced an increased number of LDs and release of prostanoids (PGE_2_, PGD_2_, and TXB_2_). Neither CdrV nor CBr induced the expression of COX-1 and COX-2 by macrophages. LDs induced by both CdrV and CBr are associated to PLIN2 recruitment and expression and were shown to be dependent on COX-1, but not COX-2 activity. Moreover, PGE_2_ colocalized to CdrV- and CBr-induced LDs, revealing the role of these organelles as sites for the synthesis of prostanoids. These results evidence, for the first time, the ability of a whole snake venom to induce formation of LDs and the potential role of these organelles for the production of inflammatory mediators during envenomation by *Crotalus* snakes.

## 1. Introduction

Snake venoms of the Viperidae family are largely recognized to induce proinflammatory reactions during envenomation [1]. However, as an exception, venoms from *Crotalus* genus snakes exert potent neurotoxic effects, do not induce inflammatory responses in their victims, and have been reported as negative modulators of the inflammatory response both *in vivo* and *in vitro* experimental conditions [2–5].

*Crotalus durissus* are venomous rattlesnakes found in the Americas with many subspecies irregularly distributed throughout the continent [6]. The major toxic and lethal effects of *Crotalus durissus* ssp. venoms are associated to crotoxin, a heterodimer toxin composed by the noncovalent association of a basic subunit (CB), comprising a phospholipase A_2_ (PLA_2_), and an acidic subunit that lacks enzymatic activity known as CA or crotapotin [7–10]. The CB subunit from *Crotalus durissus terrificus* snake venom is responsible for the myotoxic and neurotoxic actions induced by crotoxin whereas the CA subunit is recognized as a chaperone of CB [11]. Besides neurotoxicity and myotoxicity, crotoxin from *C. d. terrificus* has been reported to display antibactericidal, anti-inflammatory, immunomodulatory, and antitumor effects [4]. Moreover, the PLA_2_ CB subunit was described to negatively modulate the components of inflammatory reaction, reducing the spreading and phagocytic activity of murine macrophages both in *in vivo* and in *in vitro* experimental models [12–15]. Accordingly, this subunit has been shown to induce the release of resolutive lipid mediators, such as 15-d-PGJ2, from murine macrophages, and to elicit the formation of lipid droplets (LDs) in these cells [16, 17].

Among the seven subspecies of *C. durissus* snakes recognized in Brazil, *C. d. ruruima* is the subspecies presenting the highest level of crotoxin (82.7%) [18]. The subspecies is found in the northern area of Brazil and south of Venezuela and is responsible for most of the ophidic accidents in the state of Roraima, Brazil [19], causing letal, neurotoxic, coagulant, and myotoxic effects in the victims [20]. Interestingly, the venom of *C. d. ruruima* snake is better neutralized by antibothropic than by anticrotalic serum, strongly suggesting that this venom may have some similarities with the venom of *Bothrops* genus snakes, with regard to the ability to induce inflammatory reaction, which is the major characteristic of *Bothrops* venom actions. In spite of these particular features, the biological activities displayed by CdrV and their toxins remain poorly explored specially those related to the innate immune response.

Macrophages are central cells of the immune system by their ability to recognize antigens and to release a large array of inflammatory mediators, which regulate most of the events of inflammation [21]. Under inflammatory conditions, activated macrophages present high levels of organelles termed lipid droplets (LDs) in their cytoplasm. Nowadays, these lipid inclusions are recognized as platforms for the synthesis of inflammatory lipid mediators by compartmentalizing COX-1, COX-2, and 5-lipooxygenase enzymatic systems [22]. Moreover, LDs were shown to contain proteins related to membrane trafficking, cell signaling, lipid metabolism, and structural proteins, such as perilipin 2 (PLIN2) [23]. Although *C. d. terrificus* venom and its subunit CB have their activities upon macrophages extensively explored, the actions displayed by *C. d. ruruima* venom (CdrV) and its PLA_2_ (CBr) upon these cells remain to be explored. Therefore, we herein investigated the ability of CdrV and CBr to activate macrophages with focus on (i) formation of LDs, (ii) synthesis of lipid mediators, and (iii) mechanisms involved in these effects. Implication of LDs in the synthesis of prostaglandins was also assessed.

## 2. Materials and Methods

### 2.1. Animals

Male Swiss mice (18–20 g) were obtained from Butantan Institute (São Paulo, Brazil) facilities. The animals were housed in a temperature-controlled room (22-24°C) with a 12 h light-dark cycle and received fresh water and food *ad libitum* until they were used. The present study was approved by Butantan Institute Animal Experimentation Ethics Committee (reference no. 1019/13) in accordance with the procedures laid down by the Universities Federation for Animal Welfare.

### 2.2. *Crotalus durissus ruruima* Venom and CBr

**Figure 1:**
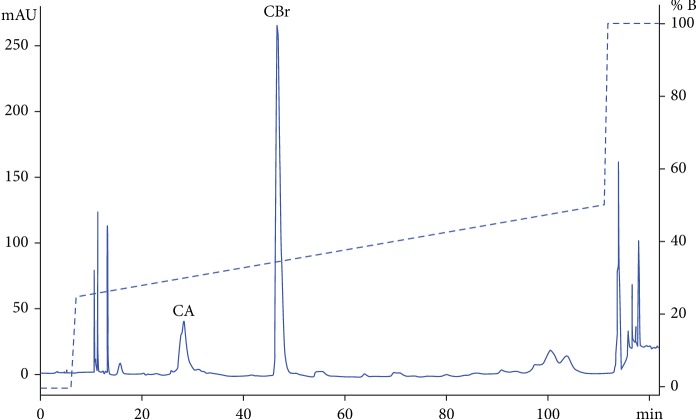
Chromatography profile of crude C. d. ruruima venom on Sephasil C8 5 *μ*m ST 4.6/250 (Amersham Biosciences). Flow rate: 1.l mL/min. Fractions of 0.5 ml were eluted under a gradient of 0.1% TFA in acetonitrile (0–25% in 0.4 mL; 25–50% in 105 mL; and 50-100% in 0.4 mL). PLA_2_ activity of CBr was 1.459 ± 0.2392 mm · mL/mg. Under the same experimental conditions, CB from C. d. terrificus gave 2.171 ± 0.1870 mm.

CdrV from the white variety was obtained from a single snake specimen by one of the authors (M.C. Dos Santos). Pharmacological properties of the crude venom were described before [24]. 2 mg of freeze-dried CdrV was loaded into a reversed-phase Sephasil C8 5 *μ*m ST 4.6/250 column (Amersham Biosciences, Buckinghamshire, UK) based on the procedure used for CB purification from *C. d. terrificus* venom [25]. CBr-containing fractions were submitted to automatic sequencing on a Shimadzu PPSQ-21A protein sequencer. The 20^th^ amino-terminal residues were HLLQFNKMIKFETRKNAIPF, which are identical to CB1 from *C. d. terrificus* venom, as previously reported [24]. PLA_2_ activity of CBr was confirmed by the clearing of a hen's egg yolk suspension incorporated into agarose gels [26]. After plotting the diameter of the clearing halos (in mm) against CBr concentration (in log scale), the enzymatic activity was expressed by the slope of the best curve fit obtained by the least square regression method. CB from *C. d. terrificus* was used as reference. CBr and CB concentrations were determined by spectrophotometry readings at 280 nm, using an extinction coefficient of 32,830 cm^−1^ M^−1^. Aliquots of CBr were freeze-dried and stored at -20°C until use.  Figure 1 presents the chromatographic profile of *C. d. ruruima* whole venom fractionation, identifying the venom major peaks, namely, crotapotin (CA) and PLA_2_ CBr. CBr peak was manually collected and submitted to automatic sequencing, confirming the identity of this PLA_2_.

### 2.3. Macrophage Harvesting

Peritoneal macrophages were obtained from the peritoneal cavity of mice 96 h after i.p. injection of 1 mL of 3% thioglycolate. The animals were killed by excess CO_2_ atmosphere, and the cells were collected by washing the peritoneal cavities with 3 mL of sterile ice-cold phosphate-buffered saline (PBS) (pH 7.2). Aliquots of the samples were used for total cell counts in a Neubauer chamber after dilution (1 : 20, *v*/*v*) in Turk's solution (0.2% crystal violet dissolved in 30% acetic acid).

Differential cell counts were performed on smears stained with Hema 3™, and more than 95% of the cell population consisted of macrophages according to conventional morphological criteria. The remaining peritoneal wash was centrifuged at 250 g for 10 minutes (20°C), and the cell pellets were used for subsequent studies after suitable dilutions.

### 2.4. Cytotoxicity Assay

Cytotoxicity of crude CdrV and CBr toward peritoneal macrophages was evaluated using a tetrazolium-based assay (MTT) and lactate dehydrogenase assay (LDH), to determinate mitochondrial functions by measuring the activity of mitochondrial enzymes and integrity of macrophage cell membrane, determining the activity of the stable and cytosolic enzyme lactate dehydrogenase, respectively [27, 28]. In brief, macrophages plated at a density of 2 × 10^5^ cells/well in 96-well plates in RPMI-1640 medium supplemented with 40 *μ*g/mL gentamicin sulfate and 2 mM L-glutamine were incubated with 100 *μ*L of selected concentrations of crude venom or CBr diluted in medium or with the same volume of medium alone (control), for 1, 3, 6, and 12 h at 37°C in a humidified atmosphere of 5% CO_2_. MTT (5 mg/mL) was dissolved in PBS and filtered to remove a small amount of insoluble residue present in some batches. Stock MTT solution (10% in culture medium) was added to all wells, and the plates were incubated at 37°C for 3 h. Next, a volume of 100 *μ*L of DMSO was added to the wells and mixed thoroughly at room temperature for 30 min. Absorbance at 540 nm was then recorded in a microtiter plate reader. Results were expressed as the percentage of viable cells, and the control cells (incubated with medium alone) were considered 100% viable. To perform LDH assay, samples were incubated with culture medium or CBr or CdrV for 1, 3, 6, and 12 h. The supernatant of the cells was transferred to a 96-well plate. After adding the LDH substrate solution to each well, the plate was incubated for 30 min. After incubation, the absorbance was read at 490 nm on an ELISA plate reader. Absorbance in wells without cells but containing the medium alone was used as the control. The percentage of cytotoxicity was calculated by the following equation: %cytotoxicity rate = [( LDH release) − (control) − (treated sample LDH activity)] ÷ total of LDH activity.

### 2.5. Macrophage Culture and Stimulation

Peritoneal macrophages were plated on sterile glass coverslips in 24-well polystyrene culture plates and allowed to attach for 30 min at 37°C under a 5% CO_2_ atmosphere. Nonadherent cells were removed by washing plates with PBS. After that, cells were challenged with selected concentrations of CdrV (1, 10, and 100 *μ*g/mL) or CBr (1.25, 3.25, 6.5, and 13 *μ*g/mL) or cultured medium alone (control) for different periods of time. Whenever appropriate, the following inhibitors were used: valeryl salicylate (30 *μ*M)—an inhibitor of COX-1—or etoricoxib (1 *μ*M)—an inhibitor of COX-2. All the stock solutions were prepared in DMSO and stored at -20°C. Aliquots were diluted in RPMI-1640 immediately before use to give the required concentration. The final DMSO concentration was always lower than 1% and had no effect on the basal number of LDs. Pharmacological inhibitors were added 60 min before the stimulation of macrophages with CdrV or CBr or culture medium (control). Cells treated with the inhibitors were analyzed for viability with the MTT assay. No significant changes in cell viability were registered with any of the above agents or vehicle at the concentrations used (Supplementary [Supplementary-material supplementary-material-1]).

### 2.6. Lipid Droplet Staining and Quantification

The analysis of LD numbers was performed in OsO_4_-stained cells as described by Leiguez et al. [17, 29]. In brief, macrophages plated on sterile glass coverslips (2 × 10^5^ cells) were fixed in 4% paraformaldehyde (PFA) in 0.1 M phosphate buffer, pH 7.2, for 15 min and stained with OsO_4_. The coverslips were washed in 0.1 M phosphate buffer, stained in 1% OsO_4_ (30 min), washed in deionized H_2_O, immersed in 1.0% thiocarbohydrazide (5 min), washed again in 0.1 M phosphate buffer, restained with 1% OsO_4_ (3 min), washed with H_2_O, and then dried and mounted. The morphology of the fixed cells was examined, and LDs were identified as round osmiophilic structures, which were counted under phase-contrast microscopy using a 100x objective lens in 50 consecutively scanned macrophages per coverslip. The total number of lipid droplets obtained in 50 macrophages is summed and divided by 50, as we can suggest an average of lipid droplets we have per cell. For assays with fluorescent-labeled LDs, peritoneal macrophages (2 × 10^5^ cells) adhered to autoclaved glass coverslips were incubated with Nile Red staining solution freshly prepared in 0.1 M phosphate buffer (10 *μ*g/mL) for 30 min at room temperature and washed with phosphate buffer. After several washes, the coverslips were mounted with Fluoromount-G and examined under a fluorescence microscope (Zeiss LSM 510 Meta confocal microscope).

### 2.7. Immunocytochemistry Analysis

Detection of PLIN2 in CdrV- or CBr-stimulated macrophages was performed by PLIN2 immunostaining. Cells were fixed in 2% formaldehyde at room temperature for 20 min and permeabilized with 0.2% Triton-X 100 in 0.1 M phosphate buffer. The unspecific sites were blocked with 0.5% albumin in 0.1 M phosphate buffer for 60 min. After that, macrophages were incubated for 1 h with a rabbit polyclonal anti-PLIN2 (1 : 2000) diluted in 0.1 M phosphate buffer with 0.2% Triton-X100. After several washes with PBS (10 min each), the preparations were incubated with secondary Alexa Fluor 488-conjugated anti-rabbit antibody (1,500) in the dark for 1 h. After new several washes, the slides were mounted with Fluoromount-G and examined under a confocal laser scanning microscope (Zeiss LSM 510 Meta). For immunodetection of PGE_2_ cells were fixed and permeabilized during 1 h at 37°C with 1% EDAC in calcium- and magnesium-free Hank's balanced salt solution (HBSS¯/¯) [30].

Then, macrophages were washed with HBSS¯/¯ and blocked with 0.5% albumin in 0.1 M phosphate buffer for 60 min. The cells were washed again with HBSS¯/¯ and incubated for 1 h with monoclonal antibodies against PGE_2_ (1 : 100). After further washes, cells were incubated with goat anti-mouse Alexa Fluor 488 (1 : 250) and Nile Red solution (1 : 250) for 1 h. The coverslips were then washed three times and mounted with Fluoromount-G and examined under a confocal laser-scanning microscope (Zeiss LSM 510 Meta) [30].

### 2.8. Western Blotting

For the analysis of COX-1, COX-2, and PLIN2 protein content, whole cell extracts were obtained by lysing the cell pellets with 80 mL of sample buffer (0.5 M Tris-HCl, pH 6.8, 20% SDS, 1% glycerol, 1 M *β*-mercaptoethanol, 0.1% bromophenol blue) and then boiled for 10 min at 100°C. Samples were resolved by electrophoresis (SDS-PAGE) on a 10% separation gel overlaid with a 5% stacking gel. After that, proteins were transferred to a nitrocellulose membrane (GE Healthcare, Buckinghamshire, UK) using a Mini Trans-Blot^®^ (Bio-Rad Laboratories, Richmond, CA, USA), and the membranes were blocked for 1 h with 5% nonfat dry milk in TTBS (20 mM Tris, 100 mM NaCl and 0.5% Tween 20). Membranes were then incubated with a primary rabbit antibody against COX-1 or COX-2 or PLIN2 (Abcam, San Francisco, USA) overnight at 4°C or with *β*-actin (Sigma, San Louis, USA) for 1 h at room temperature. They were then washed and incubated with the appropriate horseradish peroxidase-conjugated anti-rabbit IgG secondary antibody (1 : 1000 dilution, 1 h, at room temperature). Detection was by the enhanced chemiluminescence (ECL) method according to the manufacturer's instructions (GE Healthcare, Buckinghamshire, UK).

Band densities were quantified with a GS 800 Densitometer (Bio-Rad Laboratories, Richmond, CA) using Molecular Analyst® image analysis software (Bio-Rad Laboratories, Richmond, CA).

### 2.9. Quantification of PGE_2_, PGD_2_, and TXA_2_ Concentrations

PGE_2_, PGD_2_, and TXA_2_ concentrations were determined by enzyme immunoassay using a commercial kit (Cayman Chemical Company, Ann Arbor, MI). In brief, 50 *μ*L aliquots of each extracted sample were incubated with the eicosanoids conjugated with acetylcholinesterase, and the specific rabbit antiserum in 96-well plates was coated with anti-rabbit IgG mouse monoclonal antibody. After the addition of the substrate, the absorbance of the samples was recorded at 405 nm in a microplate reader (Labsystems Multiskan), and concentrations of PGE_2_, PGD_2_, and TXA_2_ concentrations were estimated from standard curves [31].

### 2.10. Statistical Analysis

Data are expressed as the mean ± standard error of mean (SEM) of at least three independent experiments. Multiple comparisons among groups were performed with one-way ANOVA followed by Tukey's test. Probabilities of less than 5% (*p*< 0.05) were considered statistically significant.

## 3. Results

### 3.1. CdrV and CBr Induce LD Formation in Peritoneal Macrophages

Preliminary studies were performed to evaluate the effect of CdrV and CBr on the viability of isolated macrophages by using the tetrazolium-based (MTT) and LDH assays. To this purpose, macrophages in culture were incubated with three distinct concentrations of CdrV (1, 10, and 100 *μ*g/mL) or CBr (3.25, 6.5, and 13 *μ*g/mL) or medium alone (control) for 12 h. Obtained results showed that neither CdrV nor CBr affected mitochondrial enzyme activity in comparison with control cells (data not shown). Similarly, neither CdrV nor CBr altered the release of LDH in comparison with control cells (data not shown). These results indicate that both CdrV and CBr did not affect cell viability at the concentrations and time of incubation used.

**Figure 2:**
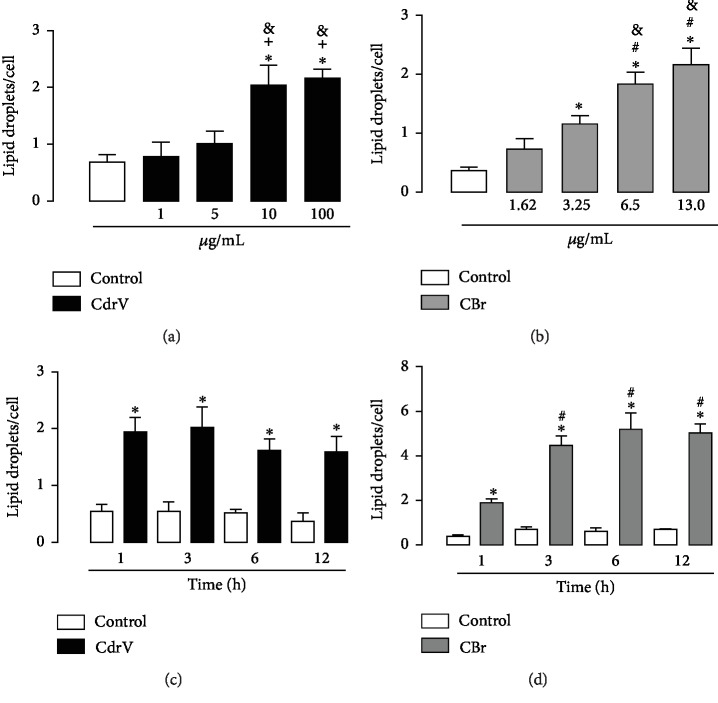
Effect of different concentrations of CdrV and CBr in LD formation in isolated macrophages. (a) Effect of CdrV and (b) CBr on LD formation in macrophages incubated with selected concentrations of CBr or RPMI (control) for 1 h. (c) Timecourse of CdrV and (d) CBr induced LD formation. Elicited macrophages were incubated with CdrV (10 *μ*g/mL), CBr (6.5 *μ*g/mL), or RPMI (control) for 1, 3, 6, or 12 h. LDs were quantified using light microscopy after OsO_4_ staining. Each bar represents the mean ± SEM of the number of LDs/cell in 50 cells. Values represent the mean ± SEM of three independent experiments with 3 to 5 animals ^∗^*p* < 0.05 compared with control cells (ANOVA).

LD formation was analyzed after incubation of macrophages with noncytotoxic concentrations of CdrV (1, 10, and 100 *μ*g/mL) or CBr (1.62, 3.25, 6.5, and 13 *μ*g/mL) or RPMI (control) for 1 h. As shown in Figures 2(a) and 2(b), incubation of macrophages with CdrV at concentrations of 10 to 100 *μ*g/mL induced a significant increase in the number of LDs compared with control cells incubated with culture medium alone. Incubation of macrophages with CBr at concentrations of 6.5 and 13 *μ*g/mL, but not 1.25 and 3.25 *μ*g/mL, induced a significant increase in the number of LDs compared with control cells.

**Figure 3:**
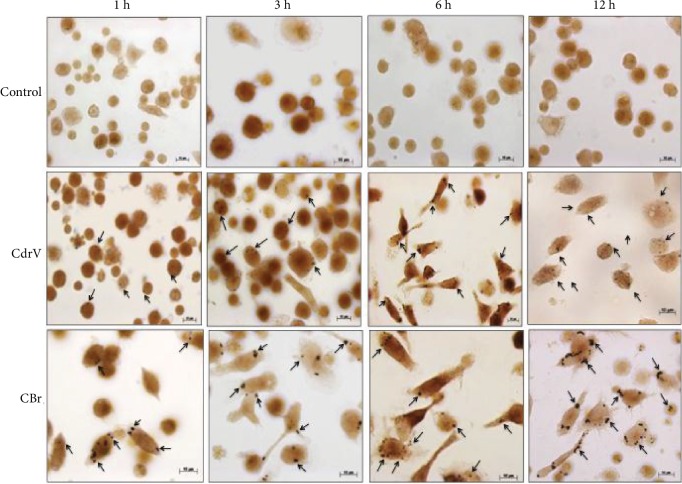
CdrV and CBr induces formation of lipid droplets in macrophages. Microphotographs of macrophages evidencing the formation of lipid droplets induced by CdrV or CBr. Elicited macrophages were collected from three independent experiments with 3 to 5 animals. Cells were placed in glass coverslips. Elicited macrophages were incubated with CdrV (10 *μ*g/mL), CBr (6.5 *μ*g/mL), or RPMI (control) for 1 to 12 h. Lipid droplets stained with osmium tetroxide are indicated by the arrows.

To determine the timecourse of CdrV- and CBr-induced LD formation, submaximal concentrations of these agents were used (10 *μ*g/mL for CdrV and 6.5 *μ*g/mL for CBr), and the number of LDs was determined after 1–12 h of incubation. As shown in Figures 2(c) and 2(d), both CdrV and CBr caused an increase in LD number from 1 up to 12 h of incubation compared with control cells. Control macrophages stained with OsO_4_ showed very few LDs in the cytoplasm. In contrast, the cytoplasm of macrophages stimulated by CdrV or CBr, for 1, 3, 6, and 12 h, was packed with LDs, which can be seen as dark punctate structures (Figure 3). Altogether, these findings indicate that both CdrV and CBr are able to induce LD biogenesis in cultured macrophages in a rapid-onset effect.

### 3.2. Both CdrV and CBr Induce PLIN2 Recruitment and Protein Expression

**Figure 4:**
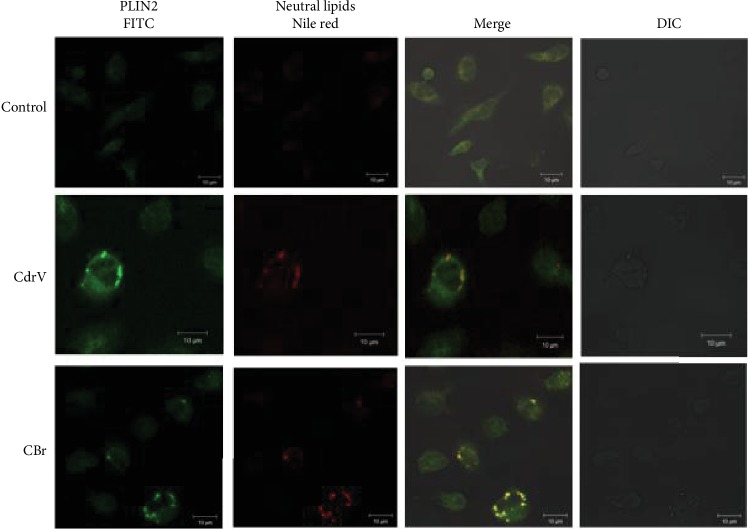
CdrV and CBr are able to induce PLIN2 recruitment in isolated macrophages. Elicited macrophages collected from three independent experiments with 3 to 5 animals were incubated with RPMI (control), CdrV (10 *μ*g/mL), or CBr (6.5 *μ*g/mL) for 3 h and labeled for LDs (with Nile Red) and for PLIN2 (with Alexa Fluor 488-conjugated antibody). The merged image shows colocalization of PLIN2 to LDs. The pictures are representative of three independent experiments.

**Figure 5:**
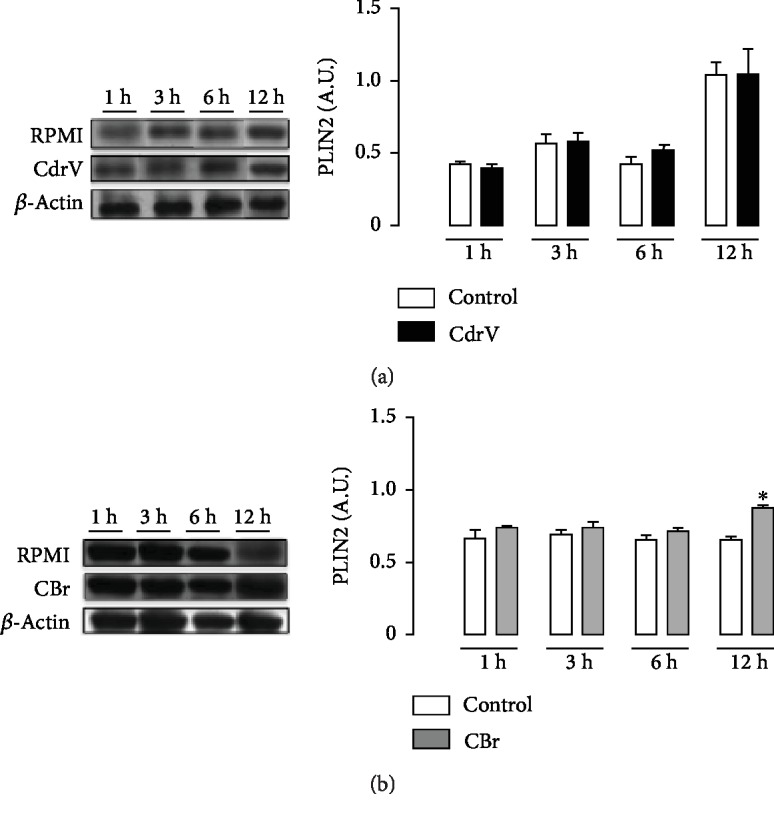
CBr but not CdrV induces an increase in PLIN2 protein expression in peritoneal macrophages. Elicited macrophages collected from three independent experiments with 3 to 5 animals were incubated with CdrV (a) (10 *μ*g/mL), CBr (b) (6.5 *μ*g/mL), or RPMI (control) for 1, 3, 6, and 12 h. Western blotting of PLIN2 and *β*-actin (loading control) in macrophage extracts and densitometric analysis of PLIN2 bands. The densities (in arbitrary units) were normalized with those of *β*-actin. Results are expressed as the mean ± SEM for three experiments. ^∗^*p* < 0.05 compared with controls.

PLIN2 plays an important role in lipid trafficking and LD assembly in macrophages and has been frequently used as a marker for LDs in leukocytes [28]. To investigate the mechanisms involved in LD formation, PLIN2 recruitment and intracellular localization were analyzed by immunofluorescence. Macrophages stimulated with CdrV (10 *μ*g/mL) or CBr (6.5 *μ*g/mL) for 3 h exhibited strong fluorescent staining (green) for PLIN2 with a punctate pattern. In unstimulated control cells, this pattern was absent. Fluorescent Nile Red-labeled neutral lipid inclusions (LDs) overlapping with stained PLIN2 were also visualized 3 h after stimulation with CdrV or CBr indicating that PLIN2 colocalizes to LDs (Figure 4). No significant staining was detected in control macrophages. Moreover, besides inducing PLIN2 recruitment, CBr, but not CdrV, induced PLIN2 expression at 12 h of incubation (Figure 5). These data demonstrate the ability of CdrV and its PLA_2_ CBr to recruit PLIN2 to form new LDs in macrophages. Moreover, the increased protein expression of PLIN2 seen under stimulation by CBr may represent an additional mechanism for LD formation induced by this PLA_2_.

### 3.3. CdrV and CBr Induce the Release of PGE_2_, PGD_2_, and TXA_2_ from Peritoneal Macrophages

**Figure 6:**
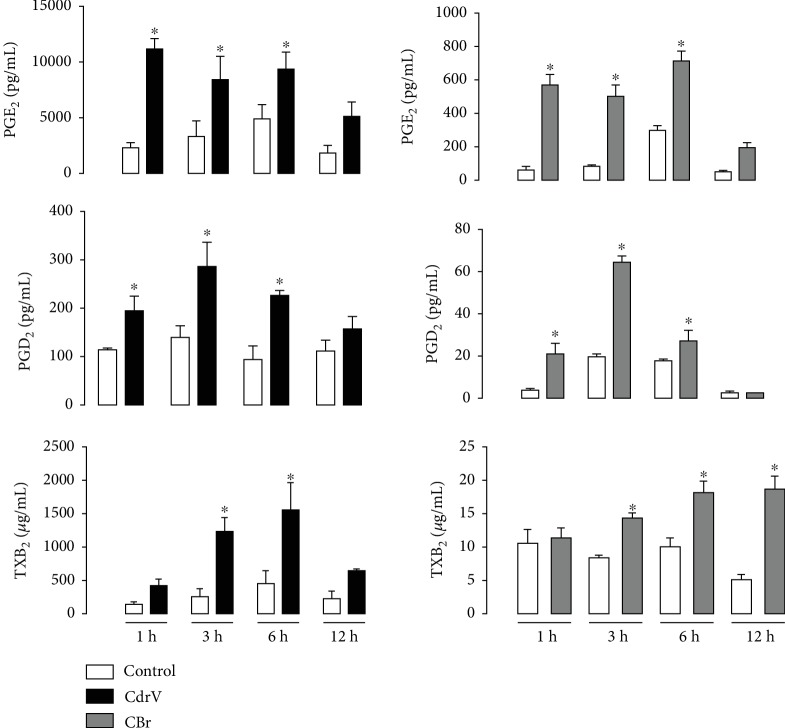
CdrV and CBr stimulate the synthesis of prostaglandins D2, E2, and thromboxane A_2_ in peritoneal macrophages. Elicited macrophages (1 × 10^6^ cells), collected from three independent experiments with 3 to 5 animals, were incubated with CdrV and CBr (6.5 *μ*g/mL) or RPMI (control) for 1 up to 12 h. PGD_2_, PGE_2_, and TXA_2_ were quantified in culture supernatants by specific EIA. Values represent the means ± SEM from 3 to 4 experiments. ^∗^*p* < 0.05 as compared with the control group.

Prostanoids are relevant mediators involved in inflammation and regulation of the immune response [32]. Moreover, one of the major interests in the study of sPLA_2_ comes from their role in the control of biosynthesis of lipid mediators in inflammatory response. We investigated the capability of CdrV and CBr to induce the release of the prostanoids PGE_2_, PGD_2_, and TXA_2_ from macrophages. As demonstrated in Figure 6, levels of PGE_2_ and PGD_2_ were significantly increased from 1 up to 6 h after stimulation of macrophages with either CdrV or CBr in comparison with respective nonstimulated controls. However, the magnitude of the CBr-induced effect was lower than that of CdrV-induced effect. With regard to TXA_2_ release, we observed an increased level of this mediator at 3 and 6 h, after stimulate cells with CdrV and after 3 to 12 h after CBr stimulus when compared with the respective controls. The effect triggered by CBr was lower than that of CdrV. These data indicate the ability of CdrV and CBr to induce production of lipid mediators by macrophages.

### 3.4. LDs Induced by CdrV and CBr Synthesize PGE_2_

**Figure 7:**
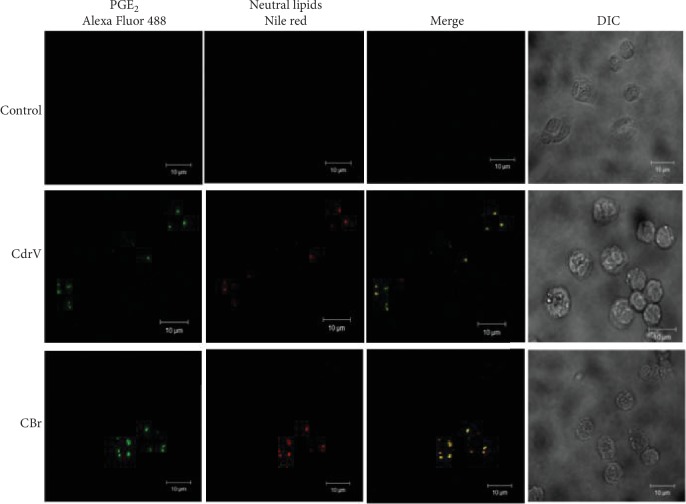
Both CdrV- and CBr-induced LDs are involved in the synthesis of PGE_2_. Elicited macrophages incubated with RPMI (control) or CdrV (10 *μ*g/mL) or CBr (6.5 *μ*g/mL) for 3 h were labeled for LDs (Nile Red) and for PGE_2_ (anti-PGE_2_ antibody). The merged image shows colocalization of PGE_2_ to LDs. The pictures are representative of three independent experiments with 3 to 5 animals.

In order to investigate the possible role of LDs as intracellular sites for the synthesis of PGE_2_, macrophages stimulated or not with CdrV or CBr were incubated with EDAC (*N*-(3-dimethylaminopropyl)-*N*′-ethylcarbodiimide hydrochloride) to immobilize the newly synthesized eicosanoids [33] and immunostained with antibodies against PGE_2_. LDs were stained with Nile Red. Macrophages stimulated with CdrV (10 *μ*g/mL) or CBr (6.5 *μ*g/mL) for 3 h exhibited a cytoplasmic staining pattern for PGE_2_ (green). This pattern was absent in the unstimulated control cells. Fluorescent Nile Red-labeled LDs were also visualized 3 h after stimulation with either CdrV or CBr and were absent in unstimulated control macrophages. Overlapping images showed that stained cytoplasmic PGE_2_ matched with LDs in CdrV- and CBr-stimulated macrophages indicating that PGE_2_ is synthesized in LDs induced by both stimuli (Figure 7).

### 3.5. CdrV and CBr Dot Not Affect COX-1 and COX-2 Protein Expression: COX-1 Is Relevant to LD Formation Induced by CdrV and CBr

**Figure 8:**
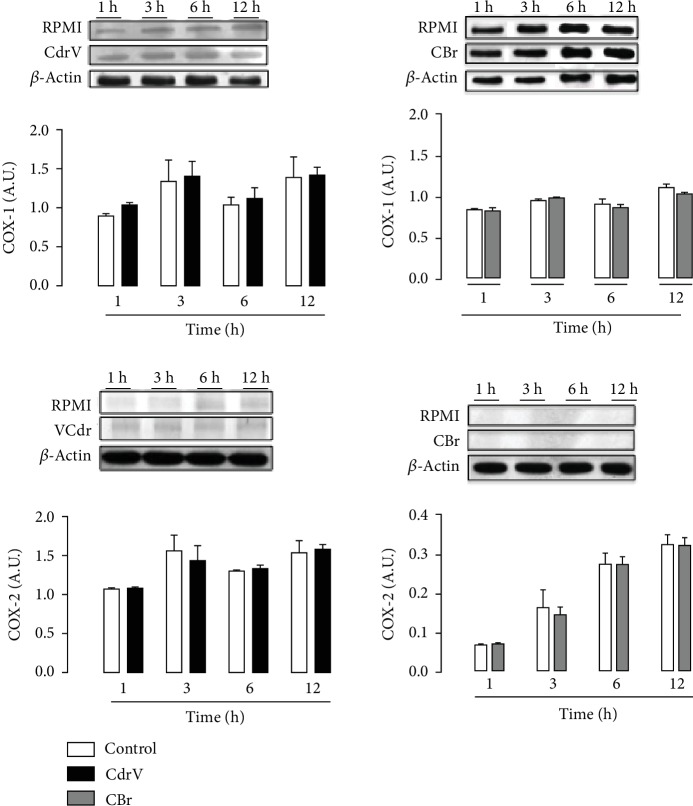
COX-2 and COX-1 protein expression is not upregulated by CdrV and CBr in peritoneal macrophages. Neither Cdr venom nor CBr affected COX-1 and COX-2 protein expression in peritoneal macrophages. Elicited macrophages from three independent experiments with 3 to 5 animals were incubated with CdrV (10 *μ*g/mL) and CBr (6.5 *μ*g/mL) or RPMI (control) for 1, 3, 6, and 12 h. The figure shows western blotting and densitometric analysis of COX-1 and COX-2 and *β*-actin (loading control) bands in macrophage extracts. The densities (in arbitrary units) were normalized with those of *β*-actin. Results are expressed as the mean ± SEM for three experiments. ^∗^*p* < 0.05 compared with controls.

**Figure 9:**
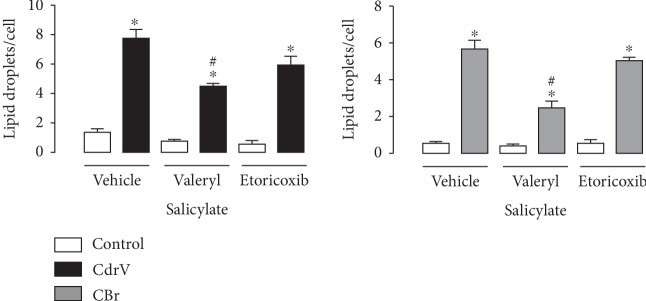
COX-1 but not COX-2 is involved in LD formation induced by CdrV and CBr in peritoneal macrophages. Elicited macrophages from three independent experiments with 3 to 5 animals were treated with valeryl salicylate (30 *μ*M) or etoricoxibe (1 *μ*M) for 1 h before stimulation with CdrV (10 *μ*g/mL) or CBr (6.5 *μ*g/mL) or RPMI (control) for 1 h. LDs were counted using light microscopy after osmium staining. Each bar represents the mean ± SEM of the number of LDs/cell in 50 cells. Values represent the means ± SEM for three to five animals. ^∗^*p* < 0.05 compared with control cells; ^#^*p* < 0.05.

Since cyclooxygenases are key enzymes in the prostaglandin biosynthetic pathway and are frequently found inside LDs, the effects of CdrV and CBr on COX-1 and COX-2 protein expression were assessed in peritoneal macrophages by western blotting analysis.  Figure 8 shows that neither CdrV nor CBr induced protein expression of COX-1 or COX-2 in comparison with controls. In addition, to verify the role of these enzymatic systems in LD formation induced by CdrV and CBr, macrophages were pretreated with valeryl salicylate (30 *μ*M), a specific COX-1 inhibitor, or with etoricoxibe (1 *μ*M), a selective inhibitor of COX-2, or their vehicles for 1 h. Pretreatment of cells with valeryl salicylate, but not with etoricoxib, significantly reduced the number of LDs induced by both CdrV and CBr in comparison with control cells, pretreated with vehicle (Figure 9). This indicates a role for COX-1-derived, but not COX-2-derived, lipid mediators in LD formation induced by both CdrV and CBr.

## 4. Discussion

In this study, the ability of CdrV and CBr to activate macrophages, a central cell in innate immune response, was evaluated. We demonstrate that CdrV and its phospholipase A_2_ CBr were able to stimulate inflammatory activities in macrophages in culture, leading to the formation of lipid droplets and biosynthesis of inflammatory lipid mediators. LD formation induced by CdrV was dependent on time and venom concentration. To the best of our knowledge, this is the first to demonstrate that a whole snake venom induces LD formation in macrophages, immunocompetent cells with key roles in inflammatory responses. This may have an impact in the pathophysiology of *C. d. ruruima*-induced envenomation and deserves further studies. The whole venom-induced effect was reproduced by CBr, its major component, thus suggesting that this PLA_2_ is responsible for the formation of LDs induced by whole CdrV. The effects observed with CBr are in agreement with the previous report of the ability of CB, a PLA_2_ from *C. d. terrificus* snake venom, to induce LDs in macrophages and reinforce the role of snake venom PLA_2_ as an important inductor of LDs biogenesis [17].

PLIN2 is the main component of perilipin family (PLIN) and considered a framework for LD formation by its capability to capture long-chain fatty acids [34]. Our results showing that PLIN2 colocalized to LDs in macrophages under stimulus either by CdrV or by CBr indicate the ability of this venom and its major component to recruit the constitutive PLIN2 from membranes. These findings may evidence a mechanism by which the whole venom and CBr increase the number of LDs in macrophages. Our data are in line with literature showing the recruitment of PLIN2 to LDs upon different inflammatory stimuli [34–36]. Moreover, CBr, but not the whole venom, upregulated PLIN2 protein expression, which correlated with the peak of LD formation. This suggests that the increased expression of PLIN2 additionally contributes to LD formation induced by this PLA_2_. These results agree with those showing that a PLA_2_ named MT-III, isolated from *Bothrops asper* snake venom, induced both PLIN2 recruitment and protein expression in macrophages [37].

Lipid mediators are widely recognized as potent modulators of inflammation. Our results showing that CdrV and CBr induced the release of PGD_2_, PGE_2_, and TXA_2_ from macrophages point to an inflammatory activity of the whole venom and its isolated component. The release of PGE_2_ and PGD_2_ occurred at the initial periods of stimulus with CdrV and CB, while the release of TXA_2_ was seen later. These differences in the course profile of the released mediators may be due to the different levels of affinity of terminal synthases to their common substrate PGH_2_ to form distinct prostanoids. This hypothesis, however, needs further confirmation [38–40]. Interestingly, our further results revealed that neither CdrV nor CBr induced the expression of COX-2, an inducible isoform during inflammatory processes. In line with these results, we found that pharmacologic inhibition of COX-1, but not COX-2 isoform, markedly reduced the release of PGE_2_. This is in accordance with previous reports that the whole venom of *C. d. terrificus* did not induce expression of COX-2, but increased the activity of COX-1 [16].

As previously mentioned, LDs are considered active organelles involved in arachidonic acid metabolism and in the synthesis of prostaglandins, as they compartmentalize the COX enzymatic system [22]. Considering that PGE_2_ is the most abundant lipid mediator produced in the early steps of inflammatory responses and has a major role in the development of edema, pain, and fever, we investigated whether LDs are involved in PGE_2_ synthesis induced by both CdrV and CBr. Our results showing LDs packed with PGE_2_ in cells stimulated either by CdrV or by CBr indicate a role of these organelles as a site of production of prostanoids upon CdrV and CBr action and strongly suggest that LDs are implicated in the inflammatory reaction displayed by the venom and its PLA_2_. Similar phenomenon was observed with a phospholipase A_2_ homologue isolated from *Bothrops asper* snake venom that exerts proinflammatory actions [37]. However, to our knowledge, this is the first demonstration of the ability of a whole venom of the *Crotalus* genus snakes to induce this phenomenon.

Considering the high intracellular levels of arachidonic acid and lipid mediators upon stimulus by whole venom and CB, we reasoned that prostanoids have a role in formation of LDs, under the present experimental condition. Our data showing that pretreatment of macrophages with COX-1 inhibitor, but not with COX-2 inhibitor, reduced LD formation induced by CdrV and CBr indicate that LD formation is dependent on prostanoids from COX-1, but not the COX-2 pathway. This event may represent part of the mechanisms involved in the formation of LDs induced in macrophages by both venom and CBr. Our data are in agreement with those of Pucer et al. [41] demonstrating the involvement of both COX-1 and COX-2 in LD formation induced by group X secreted phospholipase A_2_. In contrast to our findings, LD formation induced by CB, a PLA_2_ from *C. d. terrificus* snake venom, was not dependent on COX-derived lipid mediators [17].

## 5. Conclusion

Altogether, the obtained data allow us to conclude that CdrV and CBr in noncytotoxic concentrations are able to activate macrophages in culture inducing LD formation and production of the eicosanoids PGE_2_, PGD_2_ and TXA_2_. Prostanoids derived from COX-1, but not COX-2, are involved in LD assembly. Conversely, LDs play an active role in production of prostanoids induced by both CdrV and CBr. These data reveal a proinflammatory activity of both *Crotalus durissus ruruima* whole venom and its sPLA_2_ by exerting stimulatory activities on macrophages, which are central cells in immunological responses. Therefore, new perspectives can be opened in the search of new therapeutic targets aimed at reducing the severity of the accidents caused by the *Crotalus durissus ruruima* snake.

## Data Availability

Readers may access the data underlying the findings of this study by contacting the contributing author, Catarina Teixeira, at catarina.teixeira@butantan.gov.br.

## Supplementary Materials

Supplementary Materials Supplementary Figure 1: Effect of CdrV and CBr on cell viability. Cells were incubated with CdrV or CBr (3.25, 6.5, and 13.0 *μ*g/mL) or RPMI (control) from 1 to 12 h, and cytotoxicity was assessed by (A) LDH and (B) MTT assays. Values represent the mean ± SEM from four animals (ANOVA).
