# Supplementary material for: Crotalus durissus ruruima Snake Venom and a Phospholipase A2 Isolated from This Venom Elicit Macrophages to Form Lipid Droplets and Synthesize Inflammatory Lipid Mediators
Source: J Immunol Res. 2019 Nov 4;2019:2745286. doi: 10.1155/2019/2745286 (PMC6875421; doi:10.1155/2019/2745286)
Supplement: Supplementary Materials — Supplementary Figure 1: Effect of CdrV and CBr on cell viability. Cells were incubated with CdrV or CBr (3.25, 6.5, and 13.0 μg/mL) or RPMI (control) from 1 to 12 h, and cytotoxicity was assessed by (A) LDH and (B) MTT assays. Values represent the mean ± SEM from four animals (ANOVA). [file 2745286.f1.pdf]

Supplemmentar Figure 1:

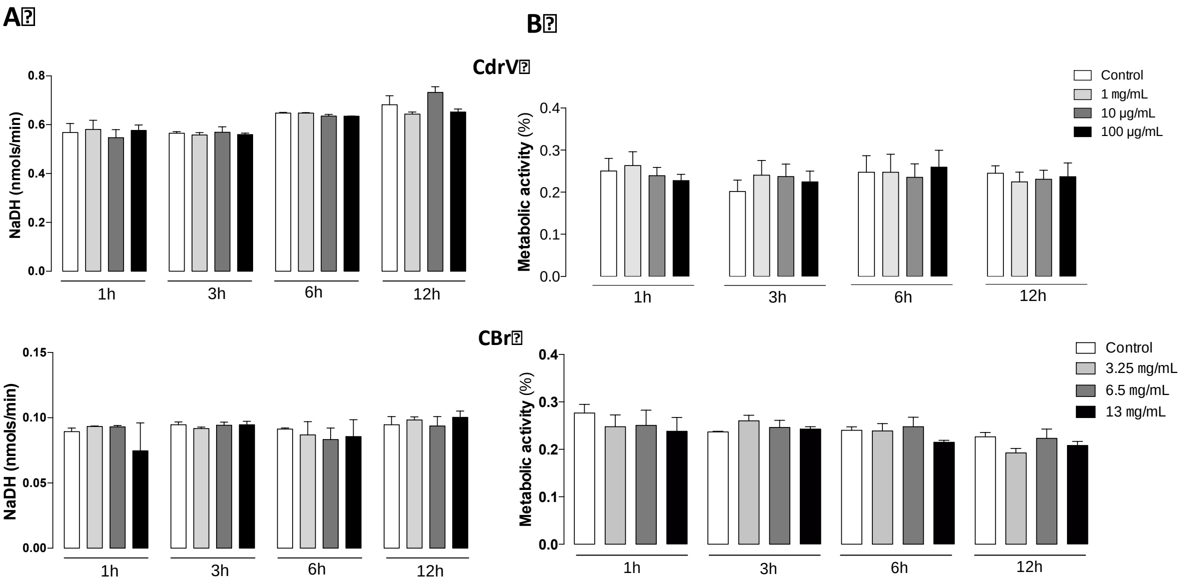

Supplemmentar Figure 1: Effect of CdrV and CBr on cell viability. Cells were incubated with CdrV or CBr (3.25, 6.5 and 13.0 µg/mL) or RPMI (control) from 1 to 12h, and cytotoxicity was assessed by **(A)** LDH **(B)** MTT assay. Values represent the mean ± SEM from four animals (ANOVA).
